# Comprehensive Clinical Characterization of Patients Diagnosed With Dilated Cardiomyopathy: A Cross-Sectional Study

**DOI:** 10.7759/cureus.66849

**Published:** 2024-08-14

**Authors:** Naresh Vishwanath Iyer Murali, Awais Ilyas Mohammed, Sumathi Gelli, Nishanth Gejjalagere Chandrashekar, Nitin Ramanathan, Rishabh Jain, Tejaswee Lohakare

**Affiliations:** 1 General Medicine, United Lincolnshire Hospitals NHS Trust, Grantham, GBR; 2 Internal Medicine, Bharat Ratna Dr. B.R. Ambedkar Medical College, Bangalore, IND; 3 General Medicine, Lincoln County Hospital, Lincoln, GBR; 4 Internal Medicine, Apollo Hospitals, Hyderabad, IND; 5 Internal Medicine, MediCiti Institute of Medical Sciences, Hyderabad, IND; 6 Emergency Medicine, United Lincolnshire Hospitals NHS Trust, Grantham, GBR; 7 Internal Medicine, Our Lady of Fatima University, Valenzuela, PHL; 8 Internal Medicine, Jagadguru Sri Shivarathreeshwara (JSS) Academy of Higher Education and Research, Mysuru, IND; 9 Internal Medicine, Cumberland Infirmary, North Cumbria Integrated Care NHS Foundation Trust, Carlisle, GBR; 10 Child Health Nursing, Srimati Radhikabai Meghe Memorial College of Nursing, Datta Meghe Institute of Higher Education and Research, Wardha, IND

**Keywords:** alcohol-related, diabetes, clinical characteristics, heart failure, dilated cardiomyopathy

## Abstract

Background

The term "cardiomyopathy" encompasses a wide range of diseases with various underlying causes. Dilated cardiomyopathy (DCM) is characterized by ventricular dilation and impaired cardiac function in the absence of congenital, valvular, hypertensive, or ischemic heart disease (IHD). This study was motivated by the high prevalence of underlying DCM and chronic heart failure, coupled with a lack of comprehensive information on DCM. The primary objective of this study was to identify the clinical characteristics and contributing factors associated with individuals diagnosed with DCM.

Methods

A total of 120 patients with DCM were enrolled in a two-year, hospital-based observational cross-sectional study conducted within the Medicine Department of a tertiary care center. The study assessed risk factors, including IHD, diabetes mellitus, alcohol consumption, and smoking.

Results

DCM was observed across all age groups, though it was notably more prevalent among middle-aged individuals (n = 50, or 41.7%) and the elderly (n = 35, or 37.5%). The condition was more commonly seen in men. IHD emerged as the predominant risk factor, affecting 75% of patients (n = 90), followed by diabetes mellitus (n = 85, or 70.8%), alcohol use (n = 75, or 62.5%), and smoking (n = 50, or 41.7%). Common symptoms included pedal edema, palpitations, easy fatigability, and exertional dyspnea.

Conclusion

In conclusion, DCM is a critical condition that necessitates vigilant monitoring. Consistent observation of symptoms, recognition of potential triggers, and prompt identification of adverse drug reactions, electrolyte disturbances, and echocardiographic changes are essential. This awareness and early detection were responsible for the lower mortality and early symptom improvement observed in the present study group.

## Introduction

Cardiomyopathies encompass a range of heart diseases that lead to structural and functional abnormalities in the heart muscle. This category includes hypertrophic cardiomyopathy (HCM), dilated cardiomyopathy (DCM), arrhythmogenic right ventricular cardiomyopathy (ARVC), and spongy heart muscle disease, many of which have genetic origins. Despite their varied causes, cardiomyopathies frequently result in heart failure due to issues with systolic or diastolic function. DCM, in particular, is characterized by the enlargement and impaired function of the ventricles, occurring without the presence of high blood pressure, valve disorders, congenital abnormalities, or coronary artery disease.

The estimated prevalence of DCM ranges from five to eight cases per 100,000 people, though these figures may underestimate the actual rates due to underreporting or undiagnosed asymptomatic cases. The clinical characteristics and progression of idiopathic DCM in adults are well-documented in the literature [1-5].

In 1980, cardiomyopathies were classified into DCM, HCM, restrictive cardiomyopathy, ARVC, and unclassified types [6,7]. In 1996, the World Health Organization, the International Society, and the Federation of Cardiology updated this classification to distinguish viral and inflammatory cardiomyopathies as separate categories [8-10].

Diagnostic criteria for DCM include a left ventricular end-diastolic size exceeding 112% of the predicted value, adjusted for age and body size, as measured by two-dimensional echocardiography. Additionally, a left ventricular ejection fraction (LVEF) below 0.45 and/or fractional shortening under 25% are critical indicators. These criteria are based on research by Henry et al. [11]. The objective of this study was to describe the clinical profile and identify risk factors in patients diagnosed with DCM.

## Materials and methods

Study design and setting

This two-year observational cross-sectional study was conducted in the Department of Medicine. Patients diagnosed with DCM were admitted through the ICU, Emergency Department, Medical Outpatient Department (OPD), or Medical Ward. The research protocol adhered to the hospital's approved ethical guidelines, as established by the Institutional Ethics Committee of MediCiti Institute of Medical Sciences (IEC-MIMS-2024-001).

Selection criteria

All patients who met the echocardiographic criteria and presented with symptoms indicative of DCM were included in this study. Individuals with systemic hypertension, coronary artery disease, congenital heart disease, or valvular heart disease were excluded.

Data sources and variables

Heart failure signs and symptoms were evaluated as part of the clinical examination. Symptoms assessed included dyspnea, palpitations, orthopnea, pedal edema, chest discomfort, cough, and easy fatigability, among others. Physical examination signs included pedal edema, hepatomegaly, elevated jugular venous pressure (JVP), basal crepitations, and heart murmurs. Baseline blood examinations were conducted by the Department of Pathology and the Department of Biochemistry. Additionally, echocardiography, electrocardiogram, and chest X-rays were performed.

Statistical analysis

Microsoft Excel (Microsoft® Corp., Redmond, WA, USA) was used to enter the data into a master file, and IBM SPSS Statistics for Windows, Version 20 (Released 2011; IBM Corp., Armonk, NY, USA) was used for analysis. Categorical and continuous variables were described using descriptive statistics, including mean, percentage, and frequency analyses. Statistical data are presented as mean ± standard deviation (SD).

## Results

Table 1 shows the demographic breakdown of participants. Out of 120 patients, 12.5% (15 patients) were between 20 and 39 years old, 41.7% (50 patients) were between 40 and 59 years old, 37.5% (45 patients) were between 60 and 79 years old, and 8.3% (10 patients) were 80 years or older. The mean age was 58 years, with an SD of 13, spanning from 20 to 90 years. Regarding gender distribution, 62.5% (75 patients) were male, and 37.5% (45 patients) were female.

**Table 1:** Demographic distribution of participants n: Number of patients; SD: Standard deviation

Demographic variable	Count (n = 120)	Percentage (%)
Age categories (years)		
20-39	15	12.50%
40-59	50	41.70%
60-79	45	37.50%
≥80	10	8.30%
Average age ± SD (range)	58 ± 13 (20-90)	
Gender distribution		
Male	75	62.50%
Female	45	37.50%

Table 2 shows the distribution of patients based on signs and symptoms and their New York Heart Association (NYHA) classification. Among the 120 patients, the following signs were observed: 79.2% (95 patients) had basal crackles, 75% (90 patients) had added heart sounds, 58.3% (70 patients) had raised JVP, 54.2% (65 patients) had apex beat shift, 41.7% (50 patients) had hepatomegaly, and 33.3% (40 patients) had an irregular pulse. Regarding symptoms, 100% (120 patients) experienced dyspnea, 75% (90 patients) had fatigue, 70.8% (85 patients) had palpitations, 66.7% (80 patients) had pedal edema, 62.5% (75 patients) had cough, 50% (60 patients) had paroxysmal nocturnal dyspnea (PND), 45.8% (55 patients) had orthopnea, 33.3% (40 patients) had chest pain, and 29.2% (35 patients) had abdominal pain. The NYHA classification showed that 8.3% (10 patients) were in Class I, 50% (60 patients) were in Class II, 33.3% (40 patients) were in Class III, and 8.3% (10 patients) were in Class IV.

**Table 2:** Allocation of patients according to their signs and symptoms, as well as their NYHA functional classification grade NYHA: New York Heart Association; JVP: Jugular venous pulse; PND: Paroxysmal nocturnal dyspnea

Clinical variable	Count (n = 120)	Percentage (%)
Signs		
Basal crackles	95	79.2%
Added heart sounds	90	75%
Raised JVP	70	58.3%
Apex beat shift	65	54.2%
Hepatomegaly	50	41.7%
Irregular pulse	40	33.3%
Symptoms		
Dyspnea	120	100%
Fatigue	90	75%
Palpitations	85	70.8%
Pedal edema	80	66.7%
Cough	75	62.5%
PND	60	50%
Orthopnea	55	45.8%
Chest pain	40	33.3%
Abdominal pain	35	29.2%
NYHA classification		
Class I	10	8.3%
Class II	60	50%
Class III	40	33.3%
Class IV	10	8.3%

Table 3 shows the distribution of patients by risk factors and comorbidities. Among the 120 patients, 75% (90 patients) had ischemic heart disease (IHD), 70.8% (85 patients) had diabetes mellitus, 62.5% (75 patients) had alcohol use, and 41.7% (50 patients) were smokers.

**Table 3:** Allocation of patients based on contributing factors and comorbid conditions

Contributing factors/comorbidity	Count (n = 120)	Percentage (%)
Ischemic heart disease (IHD)	90	75%
Diabetes mellitus	85	70.8%
Alcohol use	75	62.5%
Smoking	50	41.7%

Table 4 presents the echocardiographic findings in patients. The mean LVEF was 34.5%, with an SD of 6.1, and the values ranged from 20.0% to 48.0%. The average end-diastolic diameter was 5.8 cm with an SD of 1.2, and the range spanned from 4.0 cm to 7.0 cm. The mean end-systolic diameter was 4.6 cm with an SD of 1.1, and the measurements ranged from 3.5 cm to 5.9 cm.

**Table 4:** Echocardiographic findings in patients SD: Standard deviation

Echocardiographic parameter	Mean ± SD (range)
Left ventricular ejection fraction (%)	34.5 ± 6.1 (20.0-48.0)
End-diastolic diameter (cm)	5.8 ± 1.2 (4.0-7.0)
End-systolic diameter (cm)	4.6 ± 1.1 (3.5-5.9)

Table 5 shows the outcome of patients at discharge. Among the 120 patients, 80% (95 patients) showed improvement, while 20% (25 patients) expired. Eighty percent of patients experienced significant improvement and were discharged due to the careful and appropriate use of beta blockers, angiotensin-converting enzyme inhibitors, diuretics, blood thinners, digitalis, and other supportive treatments as needed.

**Table 5:** Outcome at discharge

Outcome	Count (n = 120)	Percentage (%)
Improved	95	80%
Expired	25	20%

## Discussion

In this study, the demographic data show that the patient population comprised 62.5% males and 37.5% females. This finding is consistent with previous studies, such as those by Teple and Kalra [12] and Dudharejia and MNandania [13], which also reported a predominance of males among patients with similar conditions. However, Kumar et al. [14] found a slightly different male-to-female ratio, reflecting variability that might be attributed to different sample populations or regional differences. The age distribution in the present study indicates that 12.5% (n = 15) of patients were aged 20-39 years, 41.7% (n = 50) were 40-59 years, 37.5% (n = 45) were 60-79 years, and 8.3% (n = 10) were 80 years or older. This distribution reveals a significant proportion of middle-aged and older patients, similar to the findings of Kumar et al. [14] and Dudharejia and MNandania [13]. The mean age of 58 years, with an SD of 13, highlights the condition's prevalence in older adults, consistent with age ranges reported in other studies.

Regarding NYHA classification, the study found that 50% (n = 60) of patients were in Class II, while 33.3% (n = 40) were in Class III. These findings suggest a predominance of moderate to severe symptoms among the study population. This distribution differs somewhat from Kumar et al. [14], who reported a higher percentage of patients in Class I. The variation might be due to differences in disease severity or stage at presentation. The symptom profile revealed that dyspnea was universally observed, with fatigue, palpitations, and pedal edema being the next most common symptoms. This symptom distribution aligns with the findings of Teple and Kalra [12] and Dudharejia and MNandania [13], who also identified dyspnea and fatigue as predominant symptoms. The high prevalence of dyspnea and fatigue underscores the debilitating nature of the condition, while the presence of pedal edema and palpitations highlights the importance of evaluating a range of symptoms in managing patients.

In terms of physical examination findings, basal crackles, abnormal heart sounds, and elevated JVP were observed in significant proportions of the study population. These findings are consistent with those reported by Dudharejia and MNandania [13], indicating advanced cardiac involvement, which aligns with the NYHA classification and symptom severity observed in this study. Echocardiographic measures showed a mean LVEF of 34.5% and significant dilation of the left ventricle, with mean end-diastolic and end-systolic diameters of 5.8 cm and 4.6 cm, respectively. These values reflect severe cardiac dysfunction, consistent with reports by Teple and Kalra [12]. The reduced LVEF and increased ventricular diameters highlight the severity of cardiac impairment in this patient population and underscore the need for effective management strategies.

At discharge, 80% (n = 95) of patients showed improvement, indicating the effectiveness of the treatment regimen, which included beta-blockers, ACE inhibitors, diuretics, anticoagulants, and digoxin. However, the 20% (n = 25) mortality rate reflects the severe nature of the condition and the challenges in managing advanced cases. This outcome emphasizes the importance of early diagnosis and comprehensive management to improve patient outcomes. Similar outcomes have been observed in the study by Mishra et al. [15], which reports comparable findings regarding patient demographics, symptom profiles, and echocardiographic measures. Overall, the present study offers significant insights into the demographic, clinical, and echocardiographic characteristics of patients with this condition. The results align with existing literature but also highlight areas of variability that warrant further investigation. The high proportion of patients showing improvement underscores the effectiveness of current treatment strategies, though the mortality rate highlights the need for ongoing research and optimization of management protocols.

Limitations of the study

Despite the valuable insights provided, several limitations should be acknowledged. The cross-sectional design of the study limits conclusions about causation and disease progression. While the sample size of 120 individuals is substantial, it may not fully represent the broader patient population, potentially affecting the generalizability of the results. The study’s reliance on clinical and echocardiographic data might not capture all aspects of disease severity and progression, as it excludes other diagnostic modalities, such as advanced imaging or biomarkers. Furthermore, the high mortality rate observed may reflect the advanced stage of the disease in the patient population studied, which could influence the overall assessment of treatment efficacy.

## Conclusions

The observations indicate that DCM is a serious condition that requires thorough monitoring. Regular and attentive observation of symptoms, vigilance for signs of vital organ dysfunction, awareness of potential symptom exacerbators, and early identification of adverse drug effects, electrolyte imbalances, and echocardiographic changes in the morphology and function of the left ventricle are crucial. The decreased mortality rate in the present study group and the early relief of symptoms can be attributed to the prompt detection and management of these factors.
